# Analysis of medication adherence and its influencing factors in outpatients with mental disorders: a mixed study in eastern of China

**DOI:** 10.1038/s41598-026-66255-0

**Published:** 2026-08-19

**Authors:** Ruoying Cheng, Wei Zhu, Zijuan Yang, Yufang Xu

**Affiliations:** 1 Nursing Department, Wuxi Higher Health Vocational Technology School, Wuxi, Jiangsu Province People’s Republic of China; 2 Psychological Department, The Mental Health Center of Jiangnan University, Wuxi, Jiangsu Province People’s Republic of China; 3 Outpatient Department, The Mental Health Center of Jiangnan University, Wuxi, Jiangsu Province People’s Republic of China

**Keywords:** Mental disorder, Medication adherence, Outpatients, Mixed study, Diseases, Health care, Medical research, Psychology, Psychology, Risk factors

## Abstract

**Background:**

Medication adherence is critical for outpatients with mental disorders, yet previous research in China has relied predominantly on quantitative surveys that have overlooked experiential and contextual factors.

**Objective:**

This mixed-methods study quantified adherence levels and associated factors, while exploring patients’ and caregivers’ perceived barriers and facilitators.

**Results:**

A total of 546 valid responses were collected (294 male, 252 female; age range 18–87 years). The sample comprised 357 (65.4%) patients with depression/anxiety, 126 (23.1%) with schizophrenia, and 63 (11.5%) with bipolar disorder. Moderate-to-high adherence (MMAS-8 score ≥ 6) was observed in 73.1% of patients. Medication adherence showed significant overall associations with education level (*χ*^2^ = 22.577, *P* = 0.007), marital status (*χ*^2^ = 5.399, *P* = 0.032), disease duration (*χ*^2 ^= 5.273, *P* = 0.037), and the relationship with the caregiver (*χ*^2^ = 7.311, *P* = 0.011). After Bonferroni correction, only education level and disease duration remained statistically significant. Five core qualitative themes were extracted: lack of illness insight, side effects, family support, doctor-patient trust, and stigma.

**Conclusions:**

Education level and disease duration are the most robust independent predictors of medication adherence in this population. Qualitative findings complement this by revealing that family support quality, stigma, and therapeutic alliance—though not captured as independent factors in the conservative statistical model—constitute essential process-level determinants that static variables fail to capture. Interventions should combine targeted health education for low-education and early-stage patients with family-based psychosocial support, stigma reduction campaigns, and shared decision-making to address both structural and relational barriers to adherence.

## Introduction

The incidence and burden of mental disorders have been increasing in recent years, and such disorders are characterized by high rates of disability, relapse and suicide. The World Health Organization (WHO) reports that nearly 1 billion people worldwide suffer from mental disorders, and one person dies by suicide every 40 s^1^. The results of a national survey of 32,552 people in China showed that the lifetime weighted prevalence of mental disorders (except dementia) was 16.6%, with anxiety disorders (7.6%) and mood disorders (7.4%) being the most prevalent, and their associated disease burden is becoming a prominent public health problem^2^. Mental disorders require ongoing treatment, primarily consisting of antipsychotic medication and psychological interventions, yet low adherence to oral medication is highly prevalent among outpatients with mental disorders.

Ongoing treatment of mental disorders is a long-term and challenging process, typically comprising antipsychotic medication and psychosocial interventions^3^. Surveys have shown that 41–61% of patients with schizophrenia^4,5^ and 20–60% of patients with bipolar disorders^6^ worldwide have poor medication adherence, and non-adherence is a major cause of relapse, reduced quality of life, and even increased risk of suicide^3,7^. The situation in China is also concerning, for example, the regular medication adherence rate among patients with severe mental disorders was only 41.78% in 2018^8^.

Previous studies on medication adherence in patients with mental disorders have mainly been quantitative analyses, focusing on factors such as individual characteristics, clinical condition, treatment regimen, and economic burden^9–11^. While quantitative studies have identified several correlates of adherence, and qualitative work has explored experiential aspects separately, these two perspectives have rarely been combined within a single study. This study therefore employed a mixed‑methods design to integrate a statistical assessment of adherence correlates with an in‑depth exploration of patients’ and caregivers’ perceived barriers and facilitators, with the aim of providing a more comprehensive understanding of adherence than quantitative surveys alone.

### Aim

This study used a mixed-methods design to examine medication adherence among outpatients with mental disorders in eastern China. A quantitative survey assessed adherence levels and identified associated demographic and clinical factors, with particular attention to adjusting for multiple comparisons to avoid spurious findings. The qualitative component explored perceived barriers and facilitators from the perspectives of patients and their primary caregivers, focusing on process‑level factors that static categorical variables cannot capture. By integrating the two forms of data, we sought to develop a fuller understanding of adherence than could be obtained from either approach alone, and to inform practical, multi‑level strategies for improving adherence.

## Methods

### Design and participants

This study was conducted at the Psychology Outpatient Clinic of Wuxi Mental Health Center, from January to December 2025. A total of 600 outpatients with mental disorders were enrolled via consecutive sampling during the study period. After excluding incomplete or invalid responses, 546 valid questionnaires were retained for analysis (91.0%).

Patient eligibility was determined according to the following criteria. Inclusion criteria were: (1) diagnosed with a mental disorder based on DSM-5^12^; (2) aged ≥ 18 years; and (3) disease duration of at least 2 years. Patients were excluded if they: (1) had been advised by their psychiatrist to discontinue medication due to serious physical illness or other contraindications; or (2) had cognitive impairment or communication difficulties that prevented informed consent.

This study was approved by the Medical Research Ethics Committee of Wuxi Higher Health Vocational Technology School (Approval Number: WXWX [20210908]) and performed in accordance with the Declaration of Helsinki. Written informed consent was obtained from all participants.

### Measures

Medication adherence was assessed using the Chinese version of the 8‑item Morisky Medication Adherence Scale (MMAS-8)^13,14^. The scale consisted of 8 items, items 1–7 were yes/no questions (with item 5 reverse‑scored); item 8 was rated on a 5‑point Likert scale (1.00 = never, 0.75 = occasionally, 0.50 = sometimes, 0.25 = often, and 0 = all the time). Total scores range from 0 to 8, with scores of 8 indicating high adherence, with 6–7 moderate adherence, and < 6 low adherence. In the Chinese version, Cronbach’s α coefficient was 0.81 and the test-retest coefficient was 0.95.

Demographic and clinical characteristics of patients and primary caregivers were collected via a structured questionnaire.

### Semi-structured interviews

Concurrently, semi‑structured interviews were conducted with 26 patients and 17 primary caregivers were randomly sampled from each adherence level (low, moderate, high) based on their MMAS-8 scores. Interviews were guided by the interview outline and scale responses. The patient interview guide mainly covered personal, disease, treatment and external environmental factors related to adherence, such as: “*How do you feel after taking the medication?*”, “*Are there any difficulties or discomfort during taking the medication?*”, “*What do you worry about taking the medication?*”, “*Do you understand the importance of medication adherence?*”. Interviews with family members focused on their understanding of long-term pharmacotherapy, difficulties encountered in helping or supervising medication taking, and the main problems and needs they faced. All interviews were conducted in a private room by a researcher and a psychiatric nurse, with each interview lasting approximately 15 min. The interviews continued until no new information emerged (i.e., data saturation was reached). The Consolidated Criteria for Reporting Qualitative Studies (COREQ) guided the design and reporting of this qualitative component^15^. To protect the privacy of participants, their names were assigned codes P1 to P26 (patients), F1 to F17 (family members). All interviews were audio‑recorded with consent and transcribed verbatim within 24 h.

### Statistical analysis

Quantitative data were analyzed using the SPSS 26.0. A chi-square test was used to examine associations between categorical variables and adherence levels. For variables with three or more categories showing significant overall results, post-hoc pairwise comparisons were performed with Bonferroni correction, and the adjusted significance threshold was set at *P* < 0.0167 (0.05/3). Qualitative data were analyzed using thematic analysis^16^. Transcripts were read repeatedly to identify initial codes, which were developed into a preliminary framework, revised iteratively, and finalized through team consensus. The research team then coded the data and consolidated themes accordingly. Analyses were conducted in Chinese; representative quotations were translated into English for reporting.

## Results

### Differences in medication adherence by demographic characteristics

Of the 546 patients, 252 (46.2%) were in the high adherence group (MMAS-8 score = 8), 147 (26.9%) in the moderate group (scores 6–7), and 147 (26.9%) in the low group (score < 6). As shown in Table 1, medication adherence differed significantly by education level, marital status, disease duration, and relationship with caregiver (all *P* < 0.05). Post-hoc pairwise comparisons with Bonferroni correction were then performed to identify specific group differences (adjusted significance threshold: *P* < 0.0167). Regarding education level, patients with high school education had significantly higher adherence than those with either primary school (*χ*² = 12.908, *P* < 0.001) or middle school (*χ*² = 6.234, *P* = 0.013) education, while no significant difference was found between middle school and primary school groups (*χ*² = 4.118, *P* = 0.042). For disease duration, patients with duration ≤ 5 years had significantly lower adherence than those with duration ≥ 11 years (*χ*² = 10.514, *P* < 0.001), but no other significant differences were observed between the remaining groups. For marital status and relationship with caregiver, no pairwise comparisons reached statistical significance after Bonferroni correction (all adjusted *P* > 0.0167).

**Table 1:** Differences in medication adherence by demographic characteristics [n (%)].

Variables	*n*	Medication Adherence			*χ* ^2^	*P*
		High	Moderate	Low		
Gender					1.591	0.104
Male	294	105(35.7)	126(42.9)	63(21.4)		
Female	252	84(33.3)	105(41.7)	63(25.0)		
Age (years)					2.367	0.539
< 30	126	21(16.7)	63(50.0)	42(33.3)		
30-60	231	84(36.4)	84(36.4)	63(27.2)		
> 60	189	42(22.2)	84(44.5)	63(33.3)		
Education level					22.577	0.007^**^
Primary school or below	105	42(40.0)	21(20.0)	42(40.0)		
Middle school	189	63(33.3)	84(44.5)	42(22.2)		
High school or above	252	126(50.0)	84(33.3)	42(16.7)		
Marital status					5.399	0.032^*^
Single	126	21(16.7)	42(33.3)	63(50.0)		
Married	315	105(33.3)	147(46.7)	63(20.0)		
Divorced & others	105	21(20.0)	42(40.0)	42(40.0)		
Medical payment type					2.354	0.143
Urban medical insurance	252	84(33.3)	126(50.0)	42(16.7)		
NCMS	189	63(33.3)	84(44.4)	42(22.3)		
Self-paying	105	42(40.0)	42(40.0)	21(20.0)		
Diagnosis					1.436	0.391
Depression/anxiety	357	105(29.4)	168(47.1)	84(23.5)		
Schizophrenia	126	21(16.7)	63(50.0)	42(33.3)		
Bipolar disorder	63	0(0.0)	42(66.7)	21(33.3)		
Disease duration, (years)					5.273	0.037^*^
≤ 5	168	42(25.0)	42(25.0)	84(50.0)		
6-10	252	84(33.3)	105(41.7)	63(25.0)		
≥ 11	126	42(33.3)	63(50.0)	21(16.7)		
Gender of primary caregiver						
Male	224	64(28.6)	64(28.6)	96(42.8)	1.032	0.831
Female	322	64(19.8)	129(40.1)	129(40.1)		
Relationship between caregiver & patient					7.311	0.011^*^
Parents	257	64(24.9)	129(50.2)	64(24.9)		
Spouse	192	64(33.3)	96(50.0)	32(16.7)		
Others	97	7(7.2)	61(62.9)	29(29.9)		
Age of primary caregiver (years)					1.055	0.677
< 45	99	34(34.3)	36(36.4)	29(29.3)		
45-65	321	96(29.9)	132(41.1)	93(29.0)		
> 65	126	32(25.4)	38(30.2)	56(44.4)		
Education of primary caregiver					1.264	0.498
Primary school or below	96	7(7.3)	65(67.7)	24(25.0)		
Middle school	193	32(16.6)	97(50.3)	64(33.1)		
High school or above	257	79(30.7)	92(35.8)	86(33.5)		

^*^P < 0.05; ^**^P < 0.01; ^***^P < 0.001. ^*^NCMS: New Cooperative Medical Scheme (a type of China’s universal healthcare system).

### Interview results

#### Key themes

Based on the interview outline and scale responses, in-depth interviews were conducted with 26 patients and 17 primary caregivers. Five themes were extracted: (1) Lack of illness insight; (2) Medication side effects; (3) Family support; (4) Doctor-patient trust; (5) Stigma.

#### Lack of illness insight

Many patients with mental disorders had varying levels of impaired insight. Most of them lacked proper awareness and self-assessment of their mental state and disease-related symptoms. Some did not perceive themselves as ill and refused to cooperate with treatment.

*“I had a bit of an emotional breakdown due to my divorce last year*,* and now I take medication when I am in a bad state. There is no need to keep taking medication*,* I can control myself.” (P9*,* 46 years)*.

More patients did not recognize the necessity or importance of maintenance medication for symptom control and relapse prevention. They reduced or stopped medication immediately after slight symptom improvement, or took it intermittently for other reasons. This was particularly common among first-episode patients and those with short disease duration, which is consistent with the quantitative findings that shorter illness duration was associated with lower adherence.

*“I’ve taken medication for more than half a year*,* and my condition is quite good now. I think I can stop taking my medication!” (P21*,* 27 years)*.

*“My brother has been taking medication for more than a year*,* but taking medicine every day bothers him. He feels there is no need to take it all the time*,* instead*,* he thinks it affects his health”*. *(F12*,* 67 years)*

#### Medication side effects

Almost all patients and family members mentioned the problem of medication side effects, which had a notable impact on adherence. A greater number of types and a larger total amount of medications could decrease adherence, particularly among elderly patients with comorbid chronic conditions who were taking multiple medications. Many patients reported discomfort such as dry mouth, drowsiness, dizziness and constipation after taking medication. Female patients were particularly concerned about weight gain. The combination of multiple medications could compound side effects and contribute to emotional irritability.

*“I gained a lot of weight after taking olanzapine for a period. I’m afraid that my weight will be out of control*,* so I wanted to ask my doctor if I could stop taking it.” (P11*,* 18 years)*.

*“I’ve had hypertension and hyperlipidemia for 20 years*,* and they were well controlled with medication. Last year I was diagnosed with depression*,* so I have to take more medications. I don’t feel well when I take so many tablets*,* so I stop for several days or reduce the dosage of psychotropic drugs.” (P23*,* 69 years)*.

Some respondents, especially first-time users, worried that psychotropic drugs would affect brain function. This was consistent with the quantitative finding that patients with shorter disease duration had lower adherence.

*“My daughter is going to take the College Entrance Examination soon. But I’ve heard that psychiatric drugs would affect one’s intelligence. I’m afraid that her bad state will affect her performance*,* so I reduced the dosage a little.” (F11*,* 44 years)*.

### Family support

This study suggested that family support could influence the medication adherence among outpatients. Some family members were reluctant to accept the diagnosis of mental disorders, especially parents of adolescents. Some studies also have shown that family members may encounter various difficulties in providing care for patients with mental disorders^17,18^. Many respondents reported that patients would miss taking their medicine if there was no one to remind or supervise them.

*“My teacher advised my parents to bring me here*,* but my father has always refused to admit that I have a mental disease”*. *(P2*,* 18 years)*

*“My husband reminds me to take medicine every day. I often forget it if he is not at home. I’m also confused about the dosage of different medications”. (P19*,* 57 years)*

Although Table 1 showed no significant difference between the medication adherence and type of medical expenses payment (*P* > 0.05), some patients in the interviews mentioned that long-term medication purchases placed a certain burden on their family’s finances. Other studies have also reported pressure and difficulty associated with ongoing treatment costs^19^.

*“We came from the countryside. Ever since I was diagnosed with bipolar disorder*,* the treatment alone has cost over 8000 yuan (about $1300) a year. Meanwhile*,* my two children are in middle school*,* leaving me under great financial pressure”*. *(P13*,* 32 years)*

### Doctor-patient trust

Pharmacotherapy of mental disorders is a long-term process. Patients’ medication adherence would be affected if doctors and patients do not establish mutual trust and effective communication. In addition, mutual trust is not only the patient’s trust in the doctor, but also confidence in the treatment regimen and medications.

*“I was treated in a hospital several months ago in my hometown*,* where they just gave me medicine. I wanted to communicate with the doctor regularly about my condition*,* but they just told me to take the medicine*,* so I came to this hospital to seek better treatment*.*” (P5*,* 22 years)*.

*“My daughter had anxiety attacks twice in the past month. I don’t think the drugs work well. My mother suggested that we try some folk methods*.*” (F3*,* 47 years)*.

### Stigma

Many patients and their families regarded going to a psychiatric hospital or taking psychotropic drugs as stigmatizing. This may be due to insufficient knowledge of the disease and the social prejudice against patients with mental disorders.

*“My son threw away his medicine secretly. He said the classmates would laugh at him if they knew his condition*.*” (F10*,* 41 years)*.

Some patients believed that having a mental disorder would negatively impact their social activities and career. Therefore, they refused to take their medication or hid their illness from others.

*“I’m so worried about losing my job because of the disease. I only take the medication at home. If I go on a business trip with my colleagues*,* I will have to stop the medication for a few days”*. *(P25*,* 41 years)*

*“My daughter is in a relationship. She’s afraid that her boyfriend might leave her because of the illness. Meanwhile*,* she has gained a lot of weight after taking the pill*,* so she doesn’t even take it on time lately*,* and often locks herself up at home and doesn’t go out”*. *(F1*,* 52 years)*

## Discussion

Medication adherence in outpatients with mental disorders is shaped by a range of factors, particularly given the lack of consistent supervision in community settings. This study showed that the overall adherence was 73.1%, which falls within the moderate-to-high range and is comparable to previous findings from the same region^10^, but remains below the 80% threshold recommended by expert consensus^20^. After Bonferroni correction for multiple comparisons, education level and disease duration emerged as the factors most consistently associated with adherence. Notably, these quantitative patterns were echoed in our qualitative data: patients with lower education and shorter illness duration more often voiced uncertainty about the need for continued medication, suggesting that limited health literacy and underdeveloped illness insight may underlie the observed associations. By contrast, marital status and relationship with caregiver, though significant in initial analyses, did not retain statistical significance after correction—a finding that can be further understood through our interview data.

The association between education level and medication adherence is consistent with previous studies conducted in China and elsewhere^21,22^, which reported that patients with higher education tend to have better health literacy and greater awareness of the importance of regular medication. Our finding that patients with high school education had the highest adherence–significantly higher than both primary and middle school groups–suggests a potential threshold effect, wherein a certain level of education may be necessary to adequately comprehend treatment rationale and engage in self-management. This interpretation is supported by our qualitative interviews, in which patients with lower education often described confusion about medication instructions and treatment goals (e.g., *P19: ‘I’m also confused about the dosage of different medicine’*), whereas those with higher education more frequently articulated clear reasons for maintaining regular medication. However, our result differs from a study in Ethiopia^4^, where no significant association between education and adherence was observed. This discrepancy may reflect differences in healthcare accessibility and health literacy between the two regions. In eastern China, where medical resources are relatively abundant, higher education may facilitate better communication with providers and more effective navigation of the healthcare system.

Disease duration told a complementary story. Patients with a shorter course of illness (≤ 5 years) had significantly lower adherence than those with longer illness duration (≥ 11 years), a pattern that was strongly supported by our interview data. Several first-episode participants viewed their symptom improvement as a signal to stop medication (e.g., *P21: ‘I’ve taken medication for more than half a year*,* and my condition is quite good now. I think I can stop taking my medication!‘; P9: ‘There is no need to keep taking medication*,* I can control myself’*). Such views reflect a well-documented gap in insight early in the illness trajectory^23^, and our findings confirm that this remains a substantial barrier even in an urban, relatively well-resourced setting. That said, some studies have reported no association between illness duration and adherence^24,25^. One possible explanation lies in sample composition: our cohort included a considerable proportion of first-episode patients (30.8% with duration ≤ 5 years), who may be especially prone to discontinuation due to limited experience of relapse and its consequences. This interpretation aligns with the observation that mental health services in some Asian countries have historically emphasized rehabilitation for chronic, severe cases, with less attention to early-stage patients^26^. The Chinese government has called for the establishment of a three-tier prevention system for mental health and comprehensive coverage of mental health services^27^; integrating outpatients into community management and monitoring their medication use could further support adherence in this vulnerable group. Viewed alongside our qualitative evidence, these findings point to the need for early intervention efforts that specifically target patients diagnosed within the past five years–through psychoeducation on the chronic nature of mental disorders and practical guidance on managing side effects, a concern frequently raised by our participants (e.g., *P23: ‘I don’t feel well when I take so many tablets*,* so I stop for several days’*). Marital status and relationship with caregiver, although significant in the initial chi-square tests, did not remain significant after Bonferroni adjustment. This does not necessarily mean that family factors are irrelevant; rather, the influence may be more nuanced than a simple marital or caregiver category can capture. Several patients in our interviews explicitly reported that their spouse served as the primary supervisor of daily medication (e.g., *P19: ‘My husband reminds me to take medicine every day. I often forget it if he is not at home’*), reflecting the important role of spousal involvement in maintaining regular treatment. At the same time, however, some family members appeared reluctant to accept the diagnosis or were overwhelmed by the long-term emotional and financial burden of caregiving, which in turn reduced their capacity to provide consistent support (e.g., *P2: ‘My teacher advised my parents to bring me here*,* but my father has always refused to admit that I have a mental disease’*). In a cultural context marked by strong family collectivism^18^, the availability and quality of family support may vary considerably across households, and support may also come from extended family members—potentially diluting the apparent effect of marital status per se. This indicates that the effect of marital status on adherence may be confounded by other variables, including age, socioeconomic status, and caregiver-related factors. This could partially account for the inconsistent findings reported in prior studies examining the relationship between marital status and adherence^9,10,28^. Mental illness is characterized by a long duration and a high risk of relapse, which may undermine patients’ confidence in treatment^29^, and family members are not always able to provide continuous long-term care–particularly when economic constraints create gaps in supervision^9^. Financial strain, another recurring theme in our interviews, added further difficulty, especially among low-income families^19^. Although recent policy measures, such as centralized drug procurement and the abolition of drug markups in public hospitals, have alleviated some of this burden^30^, long-term illness continues to pose economic challenges. These findings highlight the need for family-oriented support strategies that address both caregiving capacity and financial barriers, alongside continued policy attention to the economic impact of chronic mental illness.

Two additional themes from the interviews–stigma and doctor-patient trust—helped explain aspects of adherence that the quantitative data alone could not fully capture. Stigma emerged as a pervasive concern: patients and families spoke of fears of social judgment, workplace discrimination, and “loss of face,” a concept especially salient in Chinese cultural contexts where mental illness is often regarded as a source of shame^31,32^. One mother described how her son secretly threw away his medication to avoid peer ridicule (e.g., *F10: ‘He said the classmates would laugh at him if they knew his condition’*), while another patient deliberately skipped doses during business trips to hide his illness from colleagues (e.g., *P25: ‘If I go on a business trip with my colleagues*,* I will have to stop the medication for a few days’*). These accounts illustrate how stigma can undermine adherence not only by discouraging treatment-seeking for fear of discrimination, but also by shaping everyday decisions about when and where to take medication, particularly among employed or socially active patients. Such diminished self-efficacy and negative social experiences may aggravate the illness and create further barriers to consistent medication use. This pattern is consistent with research in Asian settings, where stigma-related barriers are often more pronounced than in the West^31,33^.

Doctor-patient trust also surfaced as a key factor. Several participants reported switching providers because they felt their concerns were not heard (e.g., *P5: ‘I wanted to communicate with the doctor regularly*,* but they just told me to take medicine*,* so I came to this hospital for better treatment’*). This is similar to the results of other previous studies of patients with schizophrenia or bipolar disorder^34,35^, and highlights the value of shared decision-making (SDM)^36^. Psychiatric nurses play a pivotal role in this process by educating patients about SDM tools and supporting their partnership with clinicians. SDM has been incorporated into national policy in multiple countries to foster a more patient-centered and personalized approach to care^37–40^. However, the effective implementation of SDM is also limited by various factors such as patient characteristics and medical resources. Future research should explore the influencing factors of SDM for mental disorders in depth, and develop assessment tools to improve medication adherence and treatment attitudes.

### Limitations

Several limitations of this study should be noted. First, participants were recruited from a selected district, which may limit the generalizability of our findings to other populations or settings. Additionally, qualitative data were collected and analyzed in Chinese and then translated into English, which may have resulted in subtle losses of meaning. Besides, variables such as household income, employment status, and types of antipsychotic medications were not included, and future studies with more comprehensive assessments are warranted.

### Impact statement

This paper provides insights into medication adherence among outpatients with mental disorders through a mixed-methods approach. The findings highlight the need for mental health institutions, in collaboration with government agencies, communities, and families, to implement comprehensive interventions aimed at improving adherence. Such efforts are important for alleviating symptoms, preventing relapse, and reducing the burden on patients and their families.

## Conclusions

This mixed-methods study identified multiple factors influencing medication adherence among outpatients with mental disorders. The quantitative survey found that, after Bonferroni correction, patients’ education level and disease duration were key predictors of medication adherence in this sample from Wuxi, a developed city in eastern China. The qualitative interview further revealed significant barriers, including lack of illness insight, medication side effects, inadequate family support, limited doctor-patient trust and stigma. These findings suggest that mental health care providers should consider both individual-level and family-level factors in developing interventions to improve medication adherence in this population.

## Data Availability

The datasets generated and analyzed during the current study are not publicly available due to their containing information that could compromise the privacy of research participants, but are available from the corresponding author (C RY) on reasonable request.
